# *Saccharomyces boulardii* CNCM I-745: A Non-bacterial Microorganism Used as Probiotic Agent in Supporting Treatment of Selected Diseases

**DOI:** 10.1007/s00284-020-02053-9

**Published:** 2020-05-29

**Authors:** Karolina Kaźmierczak-Siedlecka, Jakub Ruszkowski, Mateusz Fic, Marcin Folwarski, Wojciech Makarewicz

**Affiliations:** 1 Department of Surgical Oncology, Medical University of Gdansk, Mariana Smoluchowskiego 17, 80-214 Gdańsk, Poland; 2 Department of Nephrology, Transplantology and Internal Medicine, Medical University of Gdansk, Dębinki 7, 80-211 Gdańsk, Poland; 3 Department of Physiopathology, Medical University of Gdansk, Dębinki 7, 80-211 Gdańsk, Poland; 4 Department of Clinical and Molecular Biochemistry, Pomerian Medical University Szczecin, Powstańców Wielkopolskich 72, 70-11 Szczecin, Poland; 5 Department of Clinical Nutrition and Dietetics, Medical University of Gdansk, Dębinki 7, 80-211 Gdańsk, Poland

## Abstract

The yeast *Saccharomyces boulardii* CNCM I-745 is a unique, non-bacterial microorganism classified as a probiotic agent. In this review article, at first, we briefly summarized the mechanisms responsible for its probiotic properties, e.g. adhesion to and elimination of enteropathogenic microorganisms and their toxins; extracellular cleavage of pathogens’ virulent factors; trophic and anti-inflammatory effects on the intestinal mucosa. The efficacy of *S. boulardii* administration was tested in variety of human diseases. We discussed the results of *S. boulardii* CNCM I-745 use in the treatment or prevention of *Helicobacter pylori* infections, diarrhoea (*Clostridium difficile* infections, antibiotic-associated diarrhoea, and traveller’s diarrhoea), inflammatory bowel diseases, irritable bowel syndrome, candidiasis, dyslipidemia, and small intestine bacterial overgrowth in patients with multiple sclerosis. In case of limited number of studies regarding this strain, we also presented studies demonstrating properties and efficacy of other strains of *S. boulardii.* Administration of *S. boulardii* CNCMI I-745 during antibiotic therapy has certain advantage over bacterial probiotics, because—due to its fungal natural properties—it is intrinsically resistant to the antibiotics and cannot promote the spread of antimicrobial resistance. Even though cases of fungemia following *S. boulardii* CNCM I-745 administration were reported, it should be treated as a widely available and safe probiotic strain.

## Introduction

The human gut microbiota consists of around 1000 bacterial species [1]. The most dominant are four phyla, i.e. *Firmicutes*, *Bacteroidetes*, *Proteobacteria*, and *Actinobacteria* [1, 2]. The fungi consist of approximately < 0.1% of human gut microbiota. The composition of fungal microbiota (known as mycobiota) varies individually; in healthy individuals, the gastrointestinal mycobiota is dominated by *Candida* and *Saccharomyces* species [3]. The composition of human gut microbiota depends on many factors, e.g. life-style (eating habits, stress, the level of physical activity), administration of prebiotics, probiotics, and synbiotics as well as pharmacological therapy and surgical procedures [4]. According to Food and Agriculture Organization of United Nations and World Health Organization, probiotics are defined as “live microorganisms which when administered in adequate amounts confer a health benefit on the host” [5]. According to the International Scientific Association for Probiotics and Prebiotics, a prebiotic is “a substrate that is selectively utilized by host microorganisms conferring a health benefit” [6]. Synbiotics combine the pre- and probiotic properties [7]. The content as well as the activity of gut microbiota are important issues. The changes in gut microbiota may lead to gut dysbiosis (qualitative and quantitative alterations) [1, 2]. Infectious agents, intake of drugs (antibiotics or anti-cancer treatment) as well as inflammatory diseases are selected factors contributing to gut dysbiosis [2, 8].

Therapeutic methods, which are used to modify gut microbiota are administration of prebiotics (e.g. lactulose, fructooligosaccharides), probiotics (e.g. *Lactobacillus plantarum*, *Bifidobacterium*), synbiotics, and faecal microbiota transplantation [9, 10]. The yeast—*Saccharomyces boulardii* (*S. boulardii*) CNCM I-745 is a non-bacterial microorganism classified as a probiotic agent. It should be emphasized that *S. boulardii* CNCM I-745 is the first yeast that has been studied for use as a probiotic strain in human medicine [11]. There are available studies confirming positive effects of administration of *S. boulardii* CNCM I-745 in supporting treatment of selected diseases. This review summarizes the current knowledge about the role of *S. boulardii* in treatment of *Helicobacter pylori* infections, diarrhoea (*Clostridium difficile* infections, antibiotic-associated diarrhoea, and traveller’s diarrhoea), inflammatory bowel diseases (IBD), irritable bowel syndrome (IBS), candidiasis, dyslipidemia as well as gastrointestinal symptoms associating with small intestine bacterial overgrowth (SIBO) in multiple sclerosis (MS) patients. Most of the studies found in the literature were performed with CNCM I-745 strain, however, due to the lack of publications for some therapeutic area we also discussed other strains of *S. boulardii*.

## Properties of *S. boulardii* CNCM I-745

*Saccharomyces boulardii* CNCM I-745 was discovered by Henri Boulard (French microbiologist) in 1920 [11]. The probiotic strain of *S. boulardii* CNCM I-745 belongs to *Saccharomyces cerevisiae* species. Originally, *S. boulardii* was isolated from peels of tropical fruit. It is stable over a wide range of pH including acidic condition and temperature levels also during exposure to bile salts and gastrointestinal enzymes [12]. It is resistant to the antibiotic, because of fungal natural properties. Moreover, the administration of *S. boulardii* CNCM I-745 cannot promote antibiotic resistance because exchange of antibiotic resistance genes with bacteria is unlikely [13, 14]. In the most recent study, Moré et al. have summarized evidences that *S. boulardii* improves digestive capacity through secretion of certain enzymes (e.g. highly active sucrase), as well as through secretion of polyamines (spermine and spermidine) that increase the expression of both intestinal digestive enzymes and nutrient uptake transporters. The exact molecular mechanisms of the trophic effects of *S. boulardii* and secreted polyamines are not completely understood. They can activate at least the GRB2-SHC-CrkII-Ras-GAP-Raf-ERK1,2 pathway and the PI3K pathway, and decreased activation of p38 MAPK [15].

*Saccharomyces boulardii* CNCM I-745 shares more than 99% genomic relatedness with non-probiotic *S. cerevisiae* strains as measured by average nucleotide identity (ANI) [16]. However, there are some differentiating genetic features between *S. boulardii* and *S. cerevisiae*, such as specific microsatellite length polymorphisms of YLR177W and YKL139W genes [17, 18] and low relatedness in hybridization analysis with retrotransposon Ty917 probe [18]. Recently published comparative genomic analysis of *Saccharomyces* spp. has shown that all *S. boulardii* (including *S. boulardii* CNCM I-745) strains are distinctive from other *S. cerevisiae* due to the absence of two hexose transporters genes (HXT9 and HXT11) and two maltose-, three palatinose- and four asparagine-utilization genes (MAL11, MAL13; IMA2, IMA3, IMA4; ASP3-1, ASP3-2, ASP3-3, ASP4-4) [16]. Moreover, all sequenced *S. boulardii* strains (including CNCM I-745) possess G1278A point mutation in PGM2 gene that causes truncation of phosphoglucomutase; it impairs galactose utilization but in return confers growth advantages on glucose at a higher temperature (37 °C) [19]. Furthermore, *S. boulardii* strains, including CNCM I-745, produce higher amounts of acetic acid in 37 °C than *S*. *cerevisiae* strains [20]. The reduction in the pH of the gastrointestinal lumen is frequently described mechanism of probiotics, e.g. against *Salmonella enterica* serovar Typhimurium [21], against *Vibrio cholerae* [22] or against *Blastocystis* [23]. Using *Escherichia coli* MG1655 as an indicator strain, Offei et al. have showed that medium acidification via acetic acid results in antibacterial activity of *S*. *boulardii*, and have included unique high acetic acid production as an another probiotic properties of *S*. *boulardii* [20]. However, it requires in vivo confirmation. Interestingly, they have proved that this conspicuous difference in acetic acid production is a result of unique for *S. boulardii strains s*ingle-nucleotide polymorphisms (SNPs) in *SDH1* and *WHI2* genes [20]. To sum up, the genetic differences between *S. boulardii* CNCM I-745 and *S. cerevisiae* might provide explanation of this probiotic potency.

**Fig. 1:**
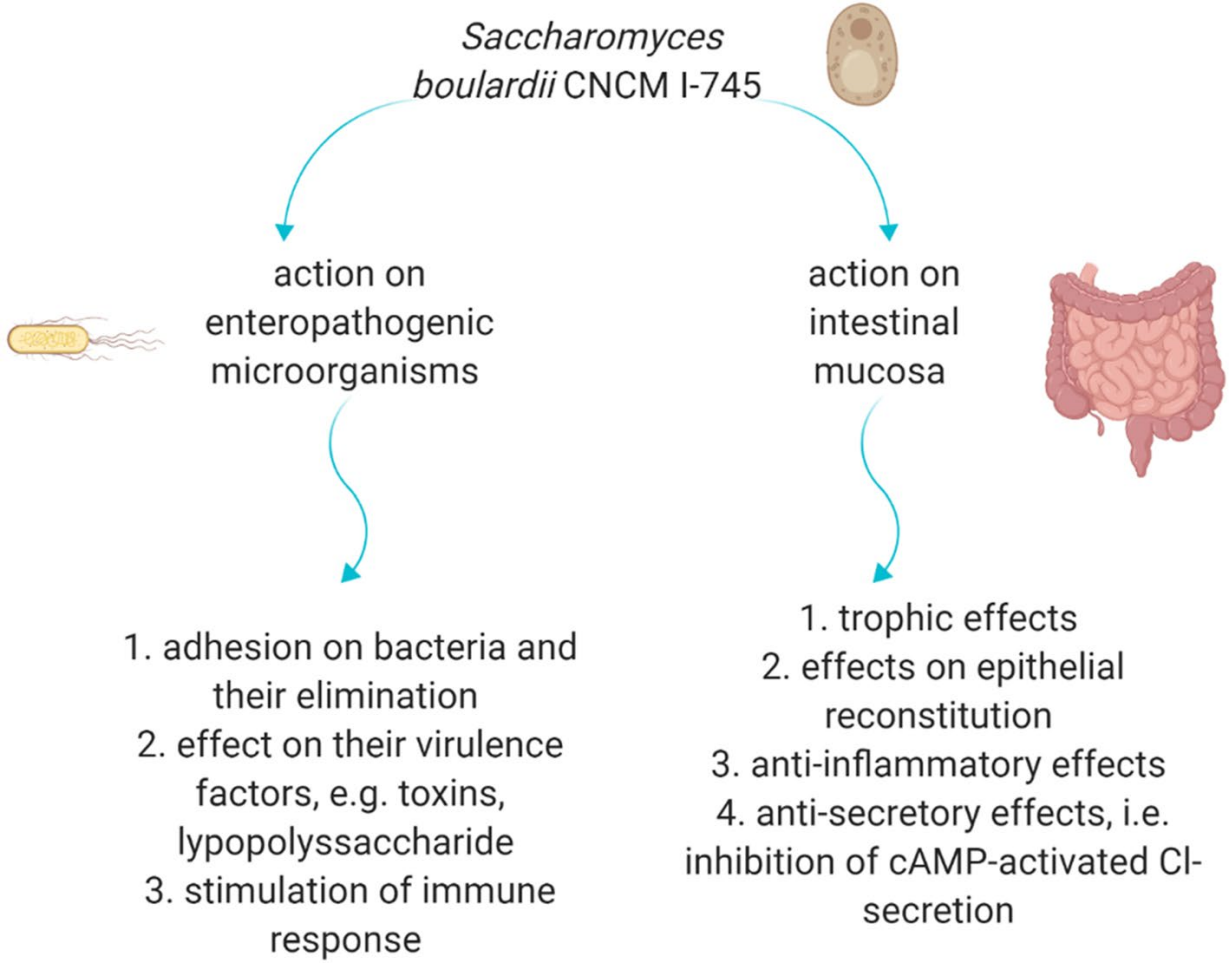
The potential mechanisms of *S. boulardii* CNCM I-745 action. *cAMP* cyclic adenosine monophosphate. Own elaboration based on literature [11, 15]

The action of *S. boulardii* CNCM I-745 is based on several mechanisms such as immunological and anti-toxin effects, pathogen-binding, as well as effects on digestive enzymes [11]. The interaction with enteropathogenic microogranisms and the effect on the intestinal mucosa are the main targets and potential mechanisms of *S. boulardii* ‘s CNCM I-745 action is presented in Fig. 1.

As it was stated above, *S. boulardii* CNCM I-745 has a beneficial effect against infections caused by pathogenic bacteria (e.g. *Clostridium difficile*, *Salmonella*, *Shigella*, *Escherichia coli*), viruses (rotavirus), and even yeasts (mainly *Candida albicans*) [11]. Using rat and mouse models, it has been demonstrated that *S. boulardii* CNCM I-745 significantly increases the concentration of intestinal sIgA and can stimulate IgA-response to *Clostridium difficile* toxin A, respectively [24, 25]. It should be emphasized that sIgA release is the first-line of defense against pathogens in the intestine [26], thus, the stimulation of sIgA after administration of *S. boulardii* CNCM I-745 is desired and may be an important mechanism for *S. boulardii*-mediated protection against diarrhoeal illnesses. Moreover, Castagliuolo et al. have reported that *S. boulardii* inhibits *Clostridium difficile* toxin A enteritis in rats by releasing a 54-kDa protease which digests the toxin A molecule and diminishes toxin A and B binding to colonic brush border membrane receptor [27]. Both toxin A and B activate the pro-inflammatory pathways (the nuclear translocation of NF-κB and the activation by phosphorylation of MAP kinases, which induce the synthesis of cytokines). It should be emphasized that cultures supernatant of *S. boulardii* inhibit pro-inflammatory cytokine (IL-8) synthesis and nuclear translocation of NF-κB. As Chen et al. reported, *S. boulardii* reduces IL-8 production via inhibition of the activation of the MAP kinases Erk1/2 and JNK/SAPK [28].

## The Effects of *S. boulardii* on Intestinal Microbiota

The oral administration of *S. boulardii* CNCM I-745 has no effect on intestinal microbiota in healthy subjects, which was shown in several studies in humans as well as in mice. However, the alterations of the microbiota in some diseases with intestinal dysbiosis after treatment with *S. boulardii* CNCM I-745 are observed [14]. The administration of *S. boulardii* CNCM I-745 can restore the intestinal microbiota faster, which was shown in a model of amoxicillin-treated mice. It was noted that the antibiotic therapy causes the increasing abundance of *Enterobacteriaceae* and *Bacteroides* and at the same time it decreases the level of *Clostridium coccoides* and *Eubacterium rectale*. The therapy of *S. boulardii* CNCM I-745 accelerated the restoration of the normal microbiota in 10 days, while the same results were observed in 22 days in untreated mice [29, 30]. In the most recent review (included preclinical and clinical data), it was confirmed that treatment with *S. boulardii* CNCM I-745 in dysbiosis leads to the faster reestablishment of a healthy microbiota [14]. However, the administration of *S. boulardii* CNCM I-745 after antibiotic therapy accelerates the recovery of the intestinal microbiota to the initial level [14]. Similarly, Swidsinski et al. reported that a total of 88% of patients with bacterial vaginosis (treated with ciprofloxacin and metronidazole) receiving *S. boulardii* CNCM I-745 quickly restored their initial individual microbial profiles [31]. Moreover, as it was stated above, *S. boulardii* is a yeast, thus it is resistant to antibiotics, which is its additional value [14, 32]. Additionally, Kelly et al. have shown that *S. boulardii* CNCM I-745 prevents the disruption of the faecal bile acids metabolism during antimicrobial therapy in healthy volunteers [33].

Moré et al. summarized evidences that *S. boulardii* CNCM I-745 support the regeneration of the intestinal microbiota after diarrhoeic dysbiosis [14]. They emphasized that the most relevant effects of this yeast on the faecal microbiota composition are the increasing of short-chain fatty acid-producing bacteria, mainly *Lachnospiraceae* and *Ruminococcaceae*, but also the increasing of *Bacteroidaceae* and *Prevotellaceae*. Additionally, suppression of pioneer bacteria was observed (they are the bacteria that have direct contact with the pellicle surface) [14, 34]. Due to anti-inflammatory, anti-secretion, pro-migratory, and adhesion effects, *S. boulardii* CNCM I-745 restores intestinal barrier function [35]. For instance, it inhibits the IL-8 secretion mediated by NF-κB and ERK1/2 phosphorylation, therefore it has anti-inflammatory properties [36]. *S. boulardii* CNCM I-745 reduces the secretion of chloride, thus it is useful in diarrhoea treatment [37]. Moreover, it restores epithelium due to restoration of glutathione and decrease in intestinal permeability [37].

## The Role of *S. boulardii* in Treatment of *Helicobacter pylori* Infection

*Helicobacter pylori* (*H. pylori*) is a Gram-negative bacterium infecting around 50% of the human population. It is one of the major factor contributing to chronic gastritis and duodenal cancer. *H. pylori* also takes part in the development of gastric cancer and mucosa-associated lymphoid tissue lymphoma (MALT) [38]. *S. boulardii* CNCM I-745 may be useful in reducing the colonization of *H. pylori* in human gastrointestinal tract in children, which was shown in Namkin et al. study [38]. This double-blind, randomized, and placebo-controlled trial included 28 asymptomatic primary school children with a positive *H. pylori* stool antigen (HpSA). The study group received one capsule daily (250 mg of lyophilized of *S. boulardii*) of *S. boulardii* CNCM I-745 per one month. There was no significant difference between groups regarding the eradication rate (*P* = 0.16). Notwithstanding, it was noted the decrease in HpSA concentration in the probiotic-treated group (*P* = 0.005 vs 0.89). According to the authors, *S. boulardii* CNCM I-745 has a positive effect on reducing *H. pylori* colonization, however, it is not capable of *H. pylori* complete eradication [38].

Additionally, another trial investigated the effect of *S. boulardii* CNCM I-745 on *H. pylori* eradication in children [38]. The study group (*n* = 102) received triple therapy (omeprazole + clarithromycin + amoxicillin or omeprazole + clarithromycin + metronidazole in case of penicillin allergy) and two sachets of *S. boulardii* CNCM I-745 (250 mg per sachet; Biocodex, Paris, France, Chinese brand name: YiHuo) orally per day for 2 weeks. The control group (*n* = 92) received only standard triple therapy. *S. boulardii* CNCM I-745 had a positive effect on prevention and treatment of diarrhoea during therapy (diarrhoea occurrence: 11.76% vs 28.26%, lasting 3.17 ± 1.08 days vs 4.05 ± 1.11 days). The 13C urea breath test confirmed eradication of *H. pylori* in 71.4% of the patients from study group and in 69.1% in the control group (not significant). This result showed that administration of this yeast only slightly increased the *H. pylori* eradication rate in comparison to standard therapy [39]. In the most recent trial of Seddik et al. [40], it was shown that *S. boulardii* CNCM I-745 plus sequential therapy for *H. pylori* infections improved *H. pylori* eradication rate (study group 86.0% vs control group 74.7%, *P* = 0.02). The addition of *S. boulardii* CNCM I-745 (250 mg ULTRA-LEVURE®, Biocodex, Morocco; twice daily per 10 days) to sequential therapy also reduced the overall incidence of treatment-associated adverse events (17.0% vs 55.7%; *P* < 0.001) and the incidence of antibiotic-associated diarrhoea (2.0% vs 46.4%; *P* = 0.02) [40].

The reduction of side effects of *H. pylori* eradication treatment after administration of *S. boulardii* was shown in a systemic review and meta-analysis [41]. It was observed that *S. boulardii* decreased side effects of *H. pylori* eradication therapy, mainly diarrhoea (RR 0.51, 95% CI 0.42–0.62; high-quality evidence) and nausea (RR 0.6, 95% CI 0.44–0.83; moderate quality of evidence). Moreover, the addition of *S. boulardii* to standard triple therapy significantly increased the eradication rate of *H. pylori*. However, it was still below the desired level of success [41]. Another study has proven that *S. boulardii* expresses neuraminidase activity selective for α2,3-linked sialic acid [42]. Sakaraya et al. showed that due to this activity, *S. boulardii* inhibits *H. pylori* adherence to duodenal epithelial cells [42].

To sum up, the administration of *S. boulardii* is rather more effective in the reduction of side effects of *H. pylori* eradication treatment than in improving *H. pylori* eradication rate. Notwithstanding, it may be used as a supporting treatment in combination with standard therapy.

## The Advantages of Using *S. boulardii* in Diarrhoea

### Antibiotic-Associated Diarrhoea

The efficiency of *S. boulardii* CNCM I-745 to reduce the incidence of diarrhoea has been investigated in Dinleyici et al. multicentre, randomised, prospective, controlled, and single-blind clinical trial including children (*n* = 363) with acute watery diarrhoea [43]. It was noted that the duration of diarrhoea was significantly reduced in group receiving *S. boulardii* CNCM I-745 (75.4 ± 33.1 vs 99.8 ± 32.5 h, *P* < 0.001) compared to control subjects. Furthermore, the mean length of emergency care unit stay was shorter with more than 19 h of difference in the *S. boulardii* CNCM I-745 group (1.20 ± 0.4 vs 2.0 ± 0.3 days, *P* < 0.001). Consequently, the administration of this probiotic strain contributes to reduction of hospital stay costs [43]. Therefore, the additional value of using *S. boulardii* CNCM I-745 for prevention antibiotic-associated diarrhoea in hospitalized patients is cost-effective observed by reducing the incidence of diarrhoea. As Vermeersch et al. reported, it means around 41.8 million euro savings for the Belgian healthcare payer [44].

Additionally, in systematic review and meta-analysis, it was confirmed that *S. boulardii* is effective in reducing the risk of antibiotic-associated diarrhoea in children as well as adults (general: 18.7% to 8.5%; children 20.9% to 8.8%; adults 17.4% to 8.2%) [45]. The daily dose of *S. boulardii* ranged from 50 to 1000 mg. The type of administered antibiotics highly varied. Diarrhoea was defined as three or more loose or watery stools per 24 h; however, the duration of diarrhoea varied from 24 h to at least 48 h. Moreover, there is no interaction between *S. boulardii* and antibiotics, thus it is also beneficial for patients [39]. However, this meta-analysis includes all strains of *S. boulardii*, not only *S. boulardii* CNCM I-745 precisely.

### *Clostridium Difficile* Infection (CDI)

*Clostridium difficile* (Gram-positive, spore-forming anaerobe) is a major cause of antibiotic-associated diarrhoea within the hospital setting [46]. One of the mechanisms leading to CDI during antimicrobial therapy is the limitation of microbial transformation of primary bile acids into secondary bile acids. Indeed, variety of in vitro studies and recently published study with animal model of antibiotic-induced CDI show that cholate-derivative primary bile acids (especially taurocholate) promote *C*. *difficile* germination and outgrowth, whereas secondary bile acids decrease spore germination and/or outgrowth [47–49]. Interestingly, Kelly et al. have reported that *S. boulardii* CNCM I-745 prevents the disruption in bile acids metabolism during antimicrobial therapy (amoxicillin plus clavulanate) in healthy volunteers, i.e. attenuates the decrease in secondary bile acid pool [50]. The results obtained by authors suggest potential mechanism of protection conferred by *S. boulardii* CNCM I-745 against post-antimicrobial CDI. Besides colonization prevention, both in vitro and animal studies demonstrated mechanisms that can lead to alleviation of CDI symptoms. Here, the cleavage of *C*. *difficile* toxin A by a 54-kDa protease released by *S. boulardii* CNCM I-745 should be mentioned again [27]. Moreover, *S. boulardii* CNCM I-745 prevents outbreak of *C. difficile*-associated cecal inflammation in hamsters, i.e. reduces cecal tissues damage, NF-κB phosphorylation, and TNF-α protein expression [51].

It should be noted that there are at least two following fields in which *S. boulardii* should be trialled in the context of CDI: prevention of primary CDI (as a probiotic taken during antibiotic treatment due to other bacterial infection) and prevention of recurrent CDI (as a probiotic taken during CDI). Three RCTs assessing the efficacy of *S*. *boulardii* CNCM I-745 in primary CDI prevention showed inconclusive results due to a low number of participants [52–55]. Surawicz et al. found that *S*. *boulardii* CNCM I-745 supplementation did neither prevent *C*. *difficile* acquisition (27% of the 81 patients receiving probiotic and 14% of the 36 patients receiving placebo acquired *C*. *difficile*; *P* = 0.18) nor significantly prevent development of CDI (2.6% of patients receiving probiotic and 7.8% of patients receiving placebo developed CDI) [53]. Similarly, McFarland et al. found that CDI was developed by 3.1 and 4.2% of patients receiving *S*. *boulardii* CNCM I-745 and placebo, respectively [54]. In a recently published RCT, Ehrhardt et al. found that only 2 of 246 patients receiving *S*. *boulardii* CNCM I-745 and 2 of 231 receiving placebo developed CDI, and due to low number of cases did not perform statistical analysis [55]. However, the administration of another *S. boulardii* strain (*S. boulardii* DBVPG 6763) was shown to be effective in the prevention of primary CDI in one-year prospective intervention study that included hospitalized patients receiving antibiotic therapy [55]. The participants (*n* = 1389) were treated with Sacchaflor (*S. boulardii* DBVPG 6763 of 5 × 10^9^) twice a day. The control groups were patients from comparable departments from other three hospitals in similar region. The reduction of CDI at the intervention hospital was observed (in the baseline CDI occurrence was 3.6%, while in the intervention period 1.5%). In the same time, CDI rates did not change at two control hospitals and decreased from 3.5 to 2.4% in the third control hospital (analysed together, occurrence decreased from 2.5 to 1.7%). These results indicate the possible role of *S. boulardii* DBVPG 6763 in CDI prevention; however, authors did not provide statistical comparison of groups [56]. In case of recurrent CDI prevention, two trials were conducted to assess efficacy of *S*. *boulardii* CNCM I-745. In McFarland et al. study, which included 124 adult patients (64 with initial CDI and 60 with history of at least one prior CDI episode), patients received standard antibiotic therapy and *S. boulardii* CNCM I-745 or placebo during 4 weeks [57]. In results, patients treated with *S. boulardii* CNCM I-745 and antibiotics had a significantly lower risk of relative risk (RR) of recurrence of CDI in comparison with those who received standard antibiotics and placebo (RR, 0.43; 95% confidence interval, 0.20 to 0.97). However, subgroup analysis revealed that the significant efficiency of *S. boulardii* CNCM I-745 was observed in patients with recurrent CDI, but not in patients with first episode of CDI [57]. Next prospective study included only patients with recurrent CDI episodes (*n* = 103) and failed to prove [58]. However, Surawicz et al., in subgroups analysis, found that *S. boulardii* CNCM I-745 significantly decreased the rate of CDI recurrence in patients receiving high-dose vancomycin (2 g/day) [58]. To sum up, the above-mentioned RCTs were underpowered to prove efficacy of *S*. *boulardii* CNCM I-745 in primary prevention of CDI (too low number of participants) and too heterogenous to find patients that would benefit from *S*. *boulardii* CNCM I-745 in the context of recurrent CDI prevention.

### Traveller’s Diarrhoea

Nowadays, traveller's diarrhoea is still a problem, especially among travellers visiting countries with low socioeconomic status and poor hygiene; it affects 20–40 million travellers per year depending on the destination and season travel [57, 59–61]. The systematic review and meta-analysis of McFarland confirmed that the administration of *S. boulardii* CNCM I-745 seems to be more effective in reducing the incidence of traveller’s diarrhoea (*R* = 0.79, 95% CI 0.72–0.87, *P* < 0.001) compared to intake of *Lactobacillus rhamnosus* GG (*P* = 0.08) and *Lactobacillus acidophilus* (*P* = 0.16) [61]. Moreover, the authors have been reported that *S. boulardii* CNCM I-745 was the only one probiotic strain significantly effective for the prevention of traveller’s diarrhoea and safety in adult travellers [61].

## The Use of *S. boulardii* in IBD

Alteration in intestinal permeability is the main factor underlying the pathogenesis of IBD. E-cadherin seems to be a target of signalling pathways during infections involving an increase in intestinal permeability. The reduction of expression and/or mislocation of E-cadherin in IBD patients was noted [62]. Terciolo et al. have reported that *S. boulardii* CNCM I-745 restores intestinal barrier integrity by regulation of E-cadherin recycling [62]; therefore, it may be useful in the prevention and treatment of IBD.

In the most recent Dong et al. trial with mouse models of colitis, the anti-inflammatory properties of *S. boulardii* were confirmed [63]. The effects of *S. boulardii* on intestinal mucosa barrier and intestinal flora were additionally assessed. The expression of zona-occludens-1 (peripheral membrane protein) and occludin (transmembrane protein) was protected by *S. boulardii* having a positive effect on inter-cellular tight junction. In addition, the level of TNF-α and IL-8 was decreased. An increase in the number of S24-7 family intestinal flora was also noted. S24-7 is an uncultured family of the order *Bacteroidales*, and it rather tends to be commensal bacteria [63]. Overall, *S. boulardii* and, as it was mentioned above, *S. boulardii* CNCM I-745 have anti-inflammatory properties as well as they have positive effect on intestinal mucosal mechanical barrier [63].

Trials assessing the role *S. boulardii* and strain *S. boulardii* CNCM I-745 in IBD prevention or treatment are still limited. Moreover, one of the major limitations of conducted studies is small sample size. Therefore, it is necessary to conduct further clinical trials (randomized, double-blind, and placebo-controlled) with larger populations.

## The Effectiveness of *S. boulardii* in IBS Treatment

One of the most important purpose of the treatment of IBS is improvement of quality of life (QOL). There are studies which confirmed positive effects of probiotic strains in the treatment of IBS. For instance, *Lactobacillus plantarum* 299v provides benefits for patients suffering from IBS, because it normalizes stool, causes relief of abdominal pain, and as a consequence improves QOL [64]. The QOL of patients with IBS may be improved after administration of *S. boulardii* CNCM I-745, which was proven in randomized, double-blind, and placebo-controlled multi-center trial [65]. The patients were divided into two groups: first receiving *S. boulardii* CNCM I-745 (*n* = 34) in daily dose of 2 × 10^11^ and second consuming placebo (*n* = 33). The overall improvement in IBS-QOL was higher in group receiving *S. boulardii* CNCM I-745 compared to participants consuming placebo (15.4% vs. 7.0%, *P* < 0.05). However, the bowel frequency and stool consistency were not changed in both groups [65].

The reduction of gastrointestinal dysfunctions in an animal model of IBS was observed after *S. boulardii* CNCM I-745 administration (in dose: 10^7^ CFU daily started 4 weeks after IG vehicle/viral inoculum and continued for 6 weeks) [66]. This probiotic yeast improved HSV-1 (Herpes Simplex Virus type 1)-induced intestinal dysmotility. The production of HSV-1-associated pro-inflammatory cytokines (TNF-α, IL-1β) in the myenteric plexus was diminished. Moreover, the level of anti-inflammatory interleukins (IL-4, IL-10) was increased [66]. The anti-inflammatory properties of *S. boulardii* CNCM I-745 were also confirmed in a randomized, double-blind, and placebo-controlled trial including patients suffering from diarrhoea-dominant IBS (*n* = 72) [67]. The *S. boulardii* CNCM I-745 was administered in a dose of 750 mg daily or placebo for 6 weeks in addition to ispaghula husk standard therapy. The level of pro-inflammatory cytokines in blood and tissue (IL-8 and TNF-α) decreased, while the anti-inflammatory cytokine (IL-10) level increased. Moreover, the increase in the IL-10/IL-12 ratio was noted in the tissue (*P* < 0.001). Furthermore, the overall QOL improvement in *S. boulardii* CNCM I-745 group was observed [67].

In McFarland et al. systematic review and meta-analysis, strain-specificity in the context of IBS has been investigated [68]. The authors found 6 randomized controlled trials (RCTs) for two similar *Saccharomyces* species in adults with IBS. Moreover, only two RCTs with *S. boulardii* CNCM I-745 had comparable outcome metrics (change in symptom scores) as the two RCTs with *Saccharomyces cerevisiae* I-3856.

To conclude, the research of *S. boulardii* CNCM I-745 and IBS shows reduction of gastrointestinal symptoms and as a consequence QOL improvement. Notwithstanding, the studies assessing the role of *S. boulardii*, mainly *S. boulardii* CNCM I-745, in IBS supporting treatment are still limited.

## The Use of *S. boulardii* in Prevention and Treatment of Candidiasis

*Candida albicans* (*C. albicans*) which is the most common opportunistic fungal pathogen isolated from human body causes superficial as well as chronic diseases [69]. The infection of *C. albicans* often develops after antibiotic treatment, while it is most serious and prevalent in immunocompromised patients. Due to increasing resistance of *C. albicans* strains to anti-fungal agents, there is a need to introduce additional therapeutic strategies. The administration of *S. boulardii* CNCM I-745 seems to be useful in these cases. The capric acid secreted by *S. boulardii* CNCM I-745 inhibits *C. albicans* filamentous growth, adhesion, and biofilm formation [69]. In another study, the effect of *S. boulardii* extract on SAP2 gene expression and antifungal susceptibility of *C. albicans* was assessed [70]. The extract from *S. boulardii* (0.48 mg/mL) changed the antifungal susceptibility pattern of isolated *C. albicans* to ketoconazole and itraconazole. The level of SAP2 expression significantly reduced. To conclude, *S. boulardii* CNCM I-745 may be used as an appropriate probiotic to prevent as well as treat the infection of *C. albicans* [70].

## The Association Between *S. boulardii* and dyslipidemia

*S. boulardii* has also potential effects on dyslipidemia. Briand et al. investigated the effects of this yeast on lipidemic profile and gut microbiota in a hamster hypercholesterolemic model [71]. *S. boulardii* CNCM I-745 was administered in a dose of 3 g per kg of body mass for 21 to 39 days. It was observed that intake of *S. boulardii* CNCM I-745 significantly modifies gut microbiota composition causing abundance of *Allobaculum* genus (the most modified taxon after *S. boulardii* administration). It should be emphasized that abundance of *Allobaculum* genus was correlated to variations in plasmatic lipoprotein levels and ABCG5 hepatic gene expression. *S. boulardii* CNCM I-745 seems to be useful in supportive treatment of hypercholesterolemia, but further studies are necessary to confirm these results in human body [71].

## *Saccharomyces boulardii*, SIBO and MS—is There a Link?

Due to the absence of clinical trials performed with *S. boulardii* CNCM I-745 strain in this context, this chapter refers to another *S. boulardii* strain. MS is a neurodegenerative immune-mediated inflammatory disorder, which affects at least 2.3 million people globally [72]. The link between gut microbiota and MS is observed. SIBO may affect patients with MS. The standard for SIBO diagnosis is the detection, on culture, of > 10^5^ colony‐forming units of bacteria per ml of jejunal fluid obtained by direct aspiration of jejunal contents [73]. According to Zhang et al., SIBO is high prevalent in Chinese patients with MS (45 patients of 118 were SIBO positive) [74]. Moreover, the gastrointestinal symptoms, such as constipation, bloating, and faecal incontinence were common (78% of subjects; 46.6%; 44.1%; respectively) in these cases [74]. It has been proven that some probiotic strains such as *Lactobacillus acidophilus*, *Lactobacillus casei*, *Lactobacillus fermentum*, and *Bifidobacterium bifidum* are effective in treating gastrointestinal symptoms observed in patients with MS. However, the data about the role of *S. boulardii* (unreported strain) in MS supporting treatment are still limited. Recently, Aghamohammadi et al. have published the study protocol for a double-blind randomized controlled clinical trial (*n* = 50) regarding the use of *S. boulardii* (in a dose of 10^10^ CFU daily for 4 months) in patients with multiple sclerosis (Iranian Clinical Trial Registry, IRCT20161022030424N1) [71]. They are going to assess changes in mental health by evaluating 28-item General Health Questionnaire (primary outcome), as well as secondary outcomes: quality of life, fatigue, pain, and serum levels of indices of inflammatory stress (high-sensitive C-reactive protein) and oxidative stress (malondialdehyde and total antioxidant capacity) [72].

## Safety of *S. boulardii*

The use of *S. cerevisiae*, as well as *S. boulardii* CNCM I-745 is rather considered safe. The increased amounts of *S. cerevisiae* infections have been noted in critically ill and/or immunocompromised patients [14]. *Saccharomyces* fungemia is one of the most severe complications secondary to administration of the probiotic especially in patients with severe general or intestinal disease who had an indwelling catheter [75]. Hwang et al. reported that *S. boulardii* caused rare gastrointestinal allergic reaction in infant with prior diagnosis of food protein-induced enterocolitis syndrome [76]. However, meta-analysis showed that administration of *S. boulardii* is safe and provides beneficial effects in children suffering from acute diarrhoea [77]. Furthermore, the results of the study by Sulik-Tyszka et al. indicate that despite the colonization of many oncohaematological patients with *Saccharomyces* spp., there were no cases of fungal sepsis caused by this species [78].

## Conclusion

The gut microbiota and its part mycobiome play an important role in many disease developments as well as treatments. *S. boulardii* CNCM I-745 is a medicinal yeast classified as non-bacterial probiotic agent. The properties of this yeast allow to recommend it as supportive treatment among others CDI, IBS, and *H. pylori* infection. On the contrary to bacterial probiotics, vitality of *S. boulardii* is not affected by antibiotics and therefore keeps its probiotic properties when co-administered with these drugs. This probiotic can then be considered for the treatment of antibiotic-associated diarrhoea. Notwithstanding, further studies should establish an appropriate dose and duration of administration of *S. boulardii*. Moreover, its role in the treatment of IBD or SIBO in multiple sclerosis patients requires further studies.
